# Moving Education Forward

**DOI:** 10.1371/journal.pcbi.0030019

**Published:** 2007-01-26

**Authors:** Fran Lewitter

The need to train biologists in computational methods is greater than ever before. Recently, I asked an MIT Biology Professor if his newer graduate students were more knowledgeable in bioinformatics and computational biology than in past years. He hesitated and said he needed to think more about it; there wasn't a clear answer. What is clear, however, is that most graduate students are computer savvy and are comfortable using computers for writing and searching the Web. They may also have used computational tools on the Web but with little understanding of the guts of the algorithm. It is this group of students, as well as those further along in their careers, whom we hope to reach with the publication of learned articles in the Education section of *PLoS Computational Biology.* Regardless of readers' levels of experience, we strive to provide a starting point for inquisitive minds.

The Education column of *PLoS Computational Biology* was introduced one year ago [1] with the goal to provide both practical and background information on important computational methods used to investigate interesting biological problems. During this first year, we have made strides toward this goal by publishing three excellent Education articles on diverse topics.

Our first tutorial was presented in April 2006 and was well-received, staying on the list of *PLoS Computational Biology* Top 10 articles downloaded well after its publication date. Practical Strategies for Discovering Regulatory DNA Sequence Motifs by MacIsaac and Fraenkel [2] explores microarray experiments, a common way of investigating gene expression. Identifying sequence motifs in sets of differentially regulated genes can provide insights into the mechanisms of regulation, but care must be taken to avoid the many spurious motifs that occur in genomic sequences. This first tutorial provides strategies to improve the chances of finding functional regulatory sites. The continued interest in this first of many tutorials to come demonstrates the need for high-quality tutorials for colleagues at all career levels.

“Regardless of readers' levels of experience, we strive to provide a starting point for inquisitive minds.”

The Education section has also featured two review articles addressing topics of wide ranging interest—Functional Classification Using Phylogenomic Inference by Brown and Sjolander [3] and Modularity and Dynamics of Cellular Networks by Qi and Ge [4]. The article by Brown and Sjolander reviews the field of phylogenomic inference, a field that attempts to address, in an evolutionary context, the question: “What function does this protein perform?” This is a question many scientists struggle with in their research. The possibility of using phylogenomic inference as a solution relies heavily on the quality of available tools and on available data. Brown and Sjolander provide an excellent overview of the field, including its pitfalls and successes.

In the second review article [4], Qi and Ge summarize strategies and results for analyzing the architecture and dynamics of cellular networks. Given the quantity of high-throughput data available, it is important to learn about computational tools that can be used to understand cellular networks. To this end, Qi and Ge provide an overview of current methods as well as some insight into what is to come. The topological features and dynamic properties of complex biological networks may be the underlying controls of phenotypes and behaviors of cells.

In this first issue of *PLoS Computational Biology* in 2007, we publish the first of several tutorials presented at the Intelligent Systems for Molecular Biology meeting in Fortaleza, Brazil, in August 2006 (Genomes, Browsers and Databases: Tools for Automated Data Integration across Multiple Genomes by Schattner [5]). This tutorial is particularly useful as it describes tools for facilitating automated, genome database–querying. Many important biological questions cannot be answered simply by querying one genomic location at a time. As the author argues, it is far more efficient and informative to use batch- and programmatic database–querying.

Journal plans for this coming year include tutorials on machine learning applications in biology, text mining, and computational proteomics—just to name a few topics. But to ensure we are able to provide educational materials of value to our readership, we look to the community. If you have prepared and presented a tutorial for an oral presentation, consider submitting it to *PLoS Computational Biology*. Likewise, let us know of material already on the Web that you have found useful in educating yourselves and your students. And last, we encourage readers to indicate topics of special interest and, even better, to submit articles on these topics. In your so doing, we may better serve and inspire the widening computational biology community. Please send correspondence to ploscompbiol_education@plos.org. 
